# Overview of Curcumin and Piperine Effects on Glucose Metabolism: The Case of an Insulinoma Patient’s Loss of Consciousness

**DOI:** 10.3390/ijms24076621

**Published:** 2023-04-01

**Authors:** Simona Servida, Elena Panzeri, Laura Tomaino, Giovanni Marfia, Emanuele Garzia, Giuseppe Ciniglio Appiani, Gianluca Moroncini, Vito De Gennaro Colonna, Carlo La Vecchia, Luisella Vigna

**Affiliations:** 1 Obesity and Work Centre, Occupational Medicine Unit, Clinica del Lavoro L. Devoto, Fondazione IRCCS Ca’ Granda Ospedale Maggiore Policlinico, 20122 Milan, Italy; 2 Independent Researcher, Nutrigenetics Consultant, London DA14 5JR, UK; 3 Postgraduate School of Emergency Medicine, Università Politecnica delle Marche, 60126 Ancona, Italy; 4 Department of Clinical and Molecular Sciences, Marche Polytechnic University, 60020 Ancona, Italy; 5 Istituto di Medicina Aerospaziale “A. Mosso”, Aeronautica Militare Italiana, 20129 Milan, Italy; 6 Department of Clinical Science and Community Health, DISSCO, Università degli Studi di Milano, 20122 Milan, Italy

**Keywords:** hypoglycemia, insulinoma, curcumin, piperine, bioavailability

## Abstract

The hypoglycemic properties of curcumin supplements in therapeutic doses are well-known and may represent a useful tool for the treatment of chronic diseases such as metabolic syndrome, insulin resistance and type 2 diabetes. The poor bioavailability of curcumin can be improved with the concomitant administration of piperine, with no severe adverse effects on glycemia reported so far in the literature. In this article, we further discuss a previously reported case of a helicopter pilot, affected by grade I obesity who, under curcumin and piperine treatment, experienced a transient loss of consciousness (TLOC), during a low-altitude flight. This episode led to a diagnosis of insulinoma, previously asymptomatic. We hypothesized that the combined effects of curcumin and piperine might have caused a severe hypoglycemic episode and subsequent TLOC. Therefore, further studies should be conducted to evaluate the safety of curcumin and piperine supplementation in subjects with impaired glucose metabolism and insulin secretion.

## 1. Introduction

Curcumin is a yellow-colored polyphenol found in the dried rhizome of *Curcuma longa*, commonly known as “turmeric”, traditionally used in China and Southeast Asia for centuries. The pharmacological properties of curcumin have received growing attention in the last few decades. Currently, it is included, as an active ingredient, in supplements claiming to have antioxidant, anti-inflammatory, antimicrobial, anti-cancer, anti-diabetic and slimming effects [1,2]. Curcumin has been demonstrated to be generally safe [1,3]. Appendix A provides a summary of bioavailability, adverse effects, plasma peak concentration and safety of curcumin and piperine as single agent and in combination in supplements.

Nevertheless, its potential therapeutic applications have been limited by its poor gastrointestinal absorption and low bioavailability, mainly due to its water insolubility and rapid metabolism and excretion [2]. Therefore, novel formulations have been proposed to improve its adsorption and/or reduce its metabolization. Among them, those containing piperine seem to increase the bioavailability, efficacy and half-life of orally administered curcumin [2,4]. The biotransformation and enzymatic degradation operated by the gut microbiota may increase the biological effects of polyphenols, including curcumin, via the formation of metabolites with specific biological proprieties [1,5]. Several studies have confirmed the hypoglycemic properties of curcumin, as a single agent or in combination with piperine, through different mechanisms of action (MoA) positively affecting glucose and insulin homeostasis [6,7]. Therefore, the possible occurrence of hypoglycemia as a side effect of curcumin supplementation in combination with piperine should be investigated.

In this article, we reconsider a clinical case in which curcumin and piperine supplementation may have acted as a catalyst for incidental diagnosis of insulinoma, after a transient loss of consciousness (TLOC). On this basis, we discuss both the MoA of curcumin in regulating glucose homeostasis and the synergic effects of piperine hypothesizing its potential side effects as a discussed strong hypoglycemic agent [7].

## 2. Summary of Case Report

As previously described [8], a 50-year-old man who worked as a helicopter rescue pilot presented a TLOC of approximately 1 min and 20 sec while in service. The incident ended with no one injured as he regained consciousness and control of the helicopter. Previously, the pilot had neither shown warning symptoms nor experienced similar episodes. He presented retrograde amnesia. His past medical history was significant for grade I obesity (BMI 30.5) and mild obstructive sleep apnea syndrome (OSAS). In the 4 months before the incident, he had been on a low-calorie diet plus curcumin and piperine supplementation (curcumin 600 mg a day and piperine 8.55 mg a day) for weight loss.

The pilot was temporarily suspended from his job pending examinations. The tests for the cardiological and neurological causes of the TLOC resulted negative. Nevertheless, blood chemistry tests revealed asymptomatic recurrent fasting hypoglycemia and elevated plasmatic levels of Chromogranin A. Fine needle aspiration guided by endoscopic ultrasonography was performed to confirm the diagnosis of pancreatic insulinoma [8]. A careful analysis of the pilot’s health status and his specific work needs was carried out. He underwent a pancreaticoduodenectomy followed by organizational interventions at his workplace. The patient was able to resume his job but under the limitation of flying “only with a safety pilot and in aircrafts with dual controls”.

Based on such evidence, we hypothesized the possibility of a severe and transient hypoglycemic event in a patient with asymptomatic insulinoma triggered by the continuous use of a commercially available supplement at a daily dose of 600 mg a day of curcumin and 8.55 mg of piperine, for a period of 4 months.

## 3. Discussion

### 3.1. Curcumin and Piperine: Structure, Metabolism, Bioavailability and Safety

**Figure 1:**
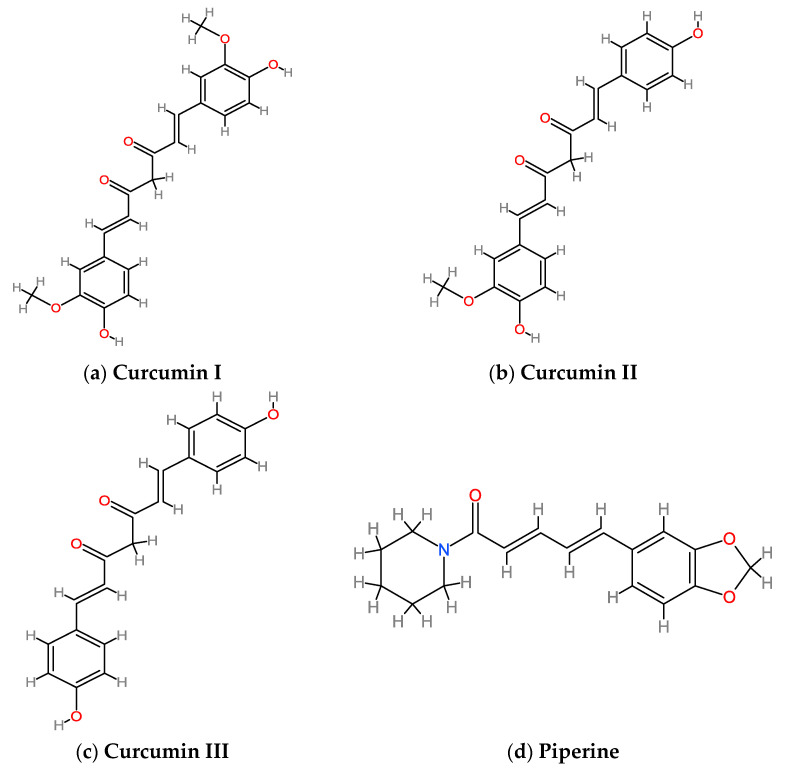
Chemical structure of curcumin and its two main analogs and piperine: (**a**) curcumin I (1E,6E)1,7-bis(4-hydroxy-3-methoxy-phenyl)-hepta-1,6-diene-3,5-dione; (**b**) curcumin II (1*E*,6*E*)-1-(4-hydroxy-3-methoxyphenyl)-7-(4-hydroxyphenyl)hepta-1,6-diene-3,5-dione; (**c**) curcumin III (Bis(4-hydroxyphenyl)-1,6-heptadiene-3,5-dione; and (**d**) piperine, (2E,4E)-5-(benzol(*d*)[1,3]dioxol-5-yl)-(piperidin-1-y)penta-2,4-dien-1-one.

Curcumin is a hydrophobic polyphenol extracted from the rhizome of *Curcuma longa* L. (turmeric), a species belonging to the *Curcuma* genus (Zingiberaceae family) [1,2]. The main constituent of curcumin extract is 1,7-bis(4-hydroxy-3-methoxyphenyl)-1,6-heptadiene-3,5-dione, a diferuloylmethane also known as curcumin I. There are, however, two other compounds known as curcumin, curcumin II (demethoxycurcumin) and curcumin III (bisdemethoxycurcumin), which differ in the number of methoxy groups on their aromatic ring (Figure 1a–c) and represent, respectively, 10–20% and 3% of the total curcuminoids [9,10]. These three curcuminoid analogs might display different pharmacological activities [11]. Experimental evidence in vitro and in vivo has shown that curcumin and its analogs exert significant anti-inflammatory and antioxidant effects [1,2], improve lipid and glucose metabolism [12], and exhibit additional beneficial biological activities, including antitumoral, neuroprotective, antimicrobial, hepatoprotective and antirheumatic ones [2,10]. Table 1 briefly summarizes the anti-diabetic activities of curcumin and piperine as single agents and in combination. 

Piperine is an alkaloid extracted from the fruits and roots of *Piper nigrum* L. (black pepper) and *Piper longum* L. (long pepper) belonging to the Piperaceae family. Piperine (Figure 1d) exists in four isomeric structures: piperine trans-trans, which displays biological activity, isopiperine cis-trans, chavicine cis-cis, and isochavicine trans-cis, which have significant anti-hepatotoxic and antioxidant effects [33,43].

The therapeutic efficacy of curcumin has been questioned because of its very low bioavailability due to poor absorption, limited tissue distribution and rapid intestinal metabolism and clearance [44]. Curcumin undergoes extensive Phase I and Phase II liver biotransformation and is quickly metabolized via glucuronidation and sulfation and then mostly eliminated through feces [45]. Animal and human studies have confirmed its limited pharmacokinetic profile [46]. Administration of single high doses of oral curcumin may cause saturation of the transport mechanism and, therefore, be counterproductive [47].

Curcumin plasma peak concentrations are usually observed at 1 to 2 h following oral administration but tend to become undetectable within 12 h [46]. New formulations have been designed to improve curcumin bioavailability including micelles, nanoparticles, liposomes, nano-emulsions and phospholipid complexes [48]. For instance, curcumin encapsulation in camel β-casein micelle can increase its solubility by 2500-fold [49]. Phytosomal curcumin also appears to be significantly more bioavailable and biologically active [50].

Although pharmacokinetics studies show improved absorption, longer half-life and higher plasma concentration of formulated curcumin, we still lack conclusive evidence regarding the comparative efficacy of different formulations [51]. In fact, the most recent research highlights the need to consider the broad range of curcuminoid metabolites, and not just unconjugated curcumin, when assessing the real bioavailability [52].

The food matrix might also play an important role in curcumin adsorption. The bioavailability of fresh or dried powdered turmeric appears to be superior compared to supplements, which are compounded with curcumin and other chemicals [53].

Recent studies have emphasized the role of intestinal microbiota in curcumin metabolism and biotransformation: it may affect its bioavailability [1,54]. Gut-derived curcumin metabolites have specific biological activities that may enhance those of the native form of this polyphenol. They could explain the incongruencies between the observed pharmacological effect of curcumin and its poor bioavailability [1]. The beneficial activities of curcumin on gut microbiota could be related to improved dysbiosis and intestinal barrier functions, increased microbial biodiversity and reduction in pro-inflammatory mediators [55]. Curcumin can modulate the ratio between beneficial and potentially pathogenic intestinal bacteria by favoring genera such as Bifidobacteria and Lactobacilli and reducing the abundance of families such as *Prevotellaceae*, *Bacteroidaceae*, *Rikenellaceae* and *Coriobacteriaceae* [1,56]. Moreover, curcumin can decrease intestinal permeability, restore barrier integrity (by preventing tight junction protein disruption [57]), increase the expression of ZO-1 and claudins and attenuate the activation of p38 MAPK [58]. Thus, some of the favorable metabolic actions of curcumin in relation to diabetes and obesity could be ascribable to its effects on microbiota [59].

The co-administration of natural inhibitors of UDP-glucuronyl transferase, such as piperine, quercetin and silybin may increase the bioavailability of oral curcumin by interfering with its glucuronidation [2]. In particular, the combination with piperine has been extensively studied and appears to significantly enhance curcumin absorption [44]. As piperine inhibits one of the enzymes responsible for the metabolization of curcumin (UDP-glucuronyl transferase), the association of the two compounds allows an increased bioavailability of curcumin as its clearance is decreased. Shoba et al. (1998) [60] showed that the co-administration of 2 g of curcumin with 20 mg of piperine to healthy individuals provoked a 2000% increase in bioavailability and much higher serum concentrations with no adverse observed effects. Studies on rats confirmed improved intestinal absorption and tissue distribution when curcumin was combined with piperine [61,62]; however, the dosages in animal studies greatly exceed those utilized in human trials.

Similarly to curcumin, the lipophilic character of piperine limits its bioavailability [18]. Piperine reaches the peak serum concentration at approximately 2–4 h after administration and remains detectable in plasma up to 48 h. It presents a secondary peak, probably due to enterohepatic recirculation of its metabolites and analogs, and a limited hepatic metabolism [63,64]. Animal studies [18] have shown that around 50% of orally administered piperine can be detected in the liver, while lower percentages are present in the heart, spleen, lungs and kidneys tissues. According to Ren et al. [63], piperine can also reach and be distributed uniformly in the brain of Caco-2 models.

Piperine is excreted in the urine in the form of numerous metabolites containing a methoxy group instead of the hydroxyl group in position 3 of the ring, and in bile after being converted into oxidized metabolites [18]. Piperine is also subjected to phase I (e.g., O-demethylation, methylation, amide hydroxylation) and phase II metabolic reactions (e.g., glucuronic acid conjugation and sulfation). Therefore, as for curcumin, its bioavailability after oral administration depends on gastrointestinal content and hepatic enzymatic biotransformation [18].

Nanoparticles can overcome the issue related to hydrophobicity and bypass its first and second metabolism. Polymer nanoparticles and liposomes appear to be the most effective in increasing piperine bioavailability [18,65].

In Kondapalli et al. (2022) [66], the administration of *Piper nigrum* extract to healthy rats enhanced the relative abundance of gut beneficial bacteria such as *Lactobacillus* and *Bifidobacterium* while decreasing *Clostridium* species.

The US Food and Drug Administration (FDA) approved curcumin as a compound “generally recognized as safe”. Similarly, JECFA (Joint FAO/WHO Expert Committee on Food Additives) and EFSA (European Food Safety Authority) have indicated, as Acceptable Daily Intake (ADI), a value of 0–3 mg/Kg for curcumin [67]. However, the European Union Herbal Monograph of *Curcuma* L. reports that curcumin may cause flatulence and gastric irritation, stimulation of bile secretion and cholangitis. Therefore, curcumin can exhibit a cholecystokinetic effect, possibly enhanced by piperine, with a contemporary risk of hepatotoxicity [68]. A few cases of hepatotoxicity induced by curcumin are reported in the literature [69,70], inducing the Italian Ministry of Health to prohibit health claims and issue a warning on curcumin-containing products [71]. To date, no severe adverse effects on glycemia have been reported.

An ADI for piperine has not been established yet, but clinical studies predict that the use of piperine, alone or in combination with other drugs, is safe at the dose of 5 mg/day with a limit of toxicity of 50 mg/kg/day [33]. Piperine may cause hemorrhagic stomach ulcers and moderate enteritis with histopathological lesions in the gastrointestinal system. Moreover, it can increase serum gonadotropins and reduce intratesticular testosterone levels in albino rats [33]. Considering the ability of piperine to influence the bioavailability of curcumin, it would be advisable to establish an ADI also for formulations containing both molecules [72].

As already mentioned, many studies evaluated the effects of piperine on the bioavailability of curcumin. The combination of the two molecules, particularly if delivered via nano-emulsions, seems to improve the absorption of curcumin [60,61,62]. A trial was carried out to evaluate the effect of combining piperine on curcumin bioavailability on albino Wistar rats and healthy human adults (curcumin 2 g/kg body weight and piperine 20 mg/kg for rats, and a dose of curcumin 2 g and piperine 20 mg for humans, respectively). The findings of the study showed that the co-administration of piperine enhanced the oral bioavailability of curcumin both in animals and humans, probably due to the piperine’s role in inhibiting the metabolism of curcumin [60]. Similar findings regarding the effects on curcumin absorption and metabolism exerted by piperine co-administration were shown by other studies on mice models [61,62]. However, Suresh and Srinivasan’s study [61] showed that the administration of both molecules did not appear to increase curcumin bioavailability in rats, as described elsewhere [72]. The supplementation of curcumin plus piperine can recover intestinal permeability and improve the antioxidant capacity in Wazhishan piglets [28]. In general, the efficacy is greater for the co-administration of curcumin plus piperine compared to high doses of curcumin or single curcumin and piperine. A human study [52] has compared curcumin adsorption after single oral administration of a standard turmeric extract, a liquid micellar preparation, a combination of piperine and curcuminoids, a phytosomal formulation and a dried colloidal suspension. The amount of adsorbed curcumin seems to be greater for the colloidal suspension than both the combination with piperine and the phytosome formulation, while no differences have been observed between piperine-curcumin preparation and turmeric dry extract. In an RCT [73], the co-administration of the two molecules resulted in an improvement of oxidative stress and inflammation in hemodialysis patients (HD).

Several mechanisms can explain the ability of piperine to increase the bioavailability of curcumin. One of these could be a non-specific action carried out at the gastric and gut level, which leads to hyperemia in the intestinal district and an increase in HCl secretion, with a subsequent increase of intestinal permeability [74]. Moreover, piperine can improve some enzymatic activities, like that of the γ-glutamyl trans-peptidase, which is involved in the intracellular transport of nutrients within enterocytes and can inhibit the drug-transporters P-glycoprotein and CYP3A4, expressed in both enterocytes and hepatocytes [68]. Another well-known mechanism is linked to piperine’s capacity to reduce curcumin glucuronidation and sulfation through a reduction in the activity of UGTs and SULTs [75].

Thus, we need further studies and more careful monitoring of patients who use supplements with a combination of curcumin and piperine, especially when there is concomitant use of other drugs [68].

### 3.2. Curcumin: Mechanism of Action on Insulin Secretion/Activity and Glycemic Homeostasis

The biological activities of insulin begin with its binding to specific Insulin Receptors (IRs) located on the plasma membrane of target cells such as those in the liver, adipose tissue, muscles and brain (although many somatic cells express IRs so insulin has pleiotropic effects) [76]. The activation of the IR, provided with tyrosine kinase activity, provokes its autophosphorylation and recruitment of several substrates, with subsequent initiation of mitogenic and metabolic downstream signaling. Most of the metabolic effects are mediated by the Insulin Receptor Substrate (IRS) family proteins, which in turn recruit phosphoinositide-3-kinase (PI3K) and then phosphoinositide-dependent kinase 1 (PDK1) and AKT [77]. Insulin signaling causes translocation and fusion of the glucose transporter GLUT 4 to the plasma membrane, which enables glucose uptake and glycogen storage in target cells (skeletal muscle and adipose tissue cells). Moreover, insulin suppresses hepatic gluconeogenesis and glycogenolysis, promotes lipid storage and suppresses lipolysis in white adipose tissue (WAT) [77]. Several in vitro and in vivo studies conducted in recent years have identified many possible MoA carried out by curcumin in regulating glucose homeostasis and insulin secretion and signaling.

#### 3.2.1. In Vitro Studies

A recent review coming from in vitro studies [4] has confirmed the positive effects of curcumin as a potential antidiabetic agent. This polyphenol was shown to improve pancreatic beta cell function and survival, increase insulin secretion and improve insulin signaling, enhance glucose uptake in adipocytes and skeletal muscle cells, inhibit gluconeogenesis and reduce lipid deposition and inflammation in liver and adipose tissue cells.

In vitro experiments conducted on skeletal muscle cells have demonstrated that curcumin treatment increases GLUT4 translocation [14,15], enhances AKT phosphorylation [20] and reduces pro-inflammatory cytokines [21]. Similar effects have been observed in adipocytes and hepatocytes [4]. However, a few studies have shown that curcumin might reduce insulin uptake and GLUT4 translocation, possibly because it interferes with AKT signaling [78,79]. Similar effects have been observed in hepatic stellate cells affected by hyperleptinemia [80]. Interestingly, Gunnik et al. (2016) [81] observed that curcumin directly inhibits glucose uptake in fibroblasts by binding to GLUT1 transporters, in an immediate, not additive and reversible way. Moreover, Priyanka et al. (2017) [17] found that curcumin treatment ameliorates hypoxic adipocytes by reducing GLUT1 expression and increasing that of GLUT4 at the same time. It has been postulated that the direct inhibition of GLUT1 and GLUT 4 caused by chronic curcumin treatment might lead to an upregulation of GLUT proteins expression as compensating mechanism. As the evidence on glucose transporters is still controversial, more studies are needed to elucidate this MoA [82]. Given the hormetic effects observed in polyphenols, it might be possible that curcumin produces a biphasic cell response based on dosage and physiological or pathological conditions [83].

Hepatocytes treated with curcumin display a reduction in gluconeogenesis and glycogenolysis accompanied by a decrease in hepatic glucose-6-phosphatase (G6Pase) and phosphoenolpyruvate carboxykinase (PEPCK) activity [22]. Rouse et al. (2014) [23] showed that the treatment of pancreatic islets with curcumin and its curcuminoid analogs resulted in increased insulin secretion and islet recovery, suggesting that it affects insulin signaling and PDE/cAMP regulation. Moreover, the above-mentioned researchers demonstrated that curcumin downregulates, in a dose-dependent manner, the mRNA expression of most of the 11 PDE (phosphodiesterase) isoenzymes, including PDE3B, PDE8A and PDE10A [23].

Additionally, curcumin may improve glucose tolerance via the stimulation of glucagon-like peptide-1 (GLP-1). The mechanism, observed in Caco-2 cells, might be mediated by inhibition of Dipeptidyl peptidase-4 (DPP IV) activity, a surface glycoprotein that degrades GLP-1 [24]. Moreover, curcumin may directly and significantly stimulate GLP-1 secretion via activation of the Ca^2+^/calmodulin-dependent kinase II pathway (independently from cAMP/PKA) [25].

Overall, the evidence from in vitro studies suggests that curcumin exerts its antidiabetic activity via multiple mechanisms that modulate both glucose uptake and insulin signaling.

#### 3.2.2. In Vivo Animal and Human Studies

In vivo studies indicate that curcumin displays remarkable antidiabetic properties by regulating glucose and lipids levels, improving insulin sensitivity and pancreatic beta-cell function, decreasing inflammation and oxidative stress and decreasing lipid peroxidation [84].

In a cohort of adult albino Wistar rats of both sexes with streptozotocin (STZ)-induced diabetes, curcumin treatment has resulted in decreased Glucose-6- phosphatase [85]. Another study on male C57BL/6 mice with STZ-induced diabetes showed that cur-cumin analog, C66, efficiently attenuated diabetic renal injury via inhibition of MAPK-mediated ACE expression and RAS activation [86]. Finally, a trial on curcumin pretreated C57/BL6J mice, which were given multiple low doses of streptozotocin to induce diabetes, showed that curcumin prevented STZ-induced diabetes, as confirmed by normoglycemia, normal glucose clearance and maintained pancreatic GLUT2 levels [87].

The evidence coming from other animal studies (diabetic KK-Ay and db/db mice) showed that curcumin significantly decreases serum glucose and HbA1c levels [26,27], with additional beneficial effects on lipids profile. Noteworthily, the administration of curcumin to db/db mice not only decreased hyperlipidemia and hyperglycemia [29] but also inhibited NLP3 inflammasome activation, showing a significant downregulation of inflammation [32].

In addition, in high-fat diet-induced diabetes models of C57BL/6J mice and albino rats, interventions with curcumin reduced circulating glucose and leptin, with a concomitant increase in adiponectin and overall improvement of insulin resistance [35,36].

It is interesting to notice that many of these research papers showed decreased insulin secretion. The dosages used in animal studies varied considerably, ranging from 0.08 mg/kg to 1500 mg/kg [84], making the efficacy comparison challenging.

Human studies seem to confirm the effects observed in those conducted on animals as curcumin has been shown to reduce serum glucose, triglyceride, LDL and HbA1c levels, as shown by a double-blind randomized clinical trial conducted on 70 type-2 diabetic (T2D) patients randomly assigned to receive curcumin (80 mg/day) or placebo for 3 months [88]. Another randomized, double-blinded, placebo-controlled trial included 240 participants with prediabetes which were assigned to receive curcumin (250 mg/day) or a placebo for 9 months. The findings showed that the intervention group presented significantly higher HOMA-β and lower C-peptide and none of the curcumin-treated participants developed T2D mellitus during the study period [41]. Similarly, another randomized, double-blinded, placebo-controlled trial carried out on 240 adult participants with T2D assigned to receive curcumin (250 mg/day) or a placebo for 6 months, showed that the intervention group presented increased serum adiponectin and decreased leptin, with an overall reduced atherogenic risk, compared to controls [42].

A recent systematic review and meta-analysis [7] have corroborated the positive effects of curcumin supplementation in improving both glycemic status and lipid profile in T2D individuals via a decreased hepatic production of glucose; it improved glucose uptake, suppressed nuclear factor-kappa B pathways and upregulated PPAR-gamma. The effects on insulin secretion are, however, not clear as some studies seem to indicate a promotion of insulin release, which is in contrast with some of the results observed in animals but aligned with those seen in vitro. More human studies are, therefore, needed to elucidate the MoA of curcumin and its analogs on glucose and insulin homeostasis, particularly to demonstrate the consequences of long-term use.

### 3.3. Piperine: Mechanism of Action on Insulin Secretion/Activity and Glycemic Homeostasis

Piperine has been demonstrated to exert various therapeutic activities: antibacterial, anticancer, antidiarrheal, antihypertensive, anti-inflammatory, antioxidant, antiparasitic and hepatoprotective [18,33]. Moreover, piperine, like curcumin, seems to have anti-diabetic properties, performed through different mechanisms of action, as confirmed by in vitro and in vivo studies.

#### 3.3.1. In Vitro Studies

Park et al. (2012) [19] showed that piperine performs its anti-diabetic activity by interacting with peroxisome proliferator-activated receptor gamma (PPAR-γ) with consequently improved insulin-sensibility in 3T3-L1 preadipocytes [19]. Moreover, the findings of a study on L6 myotubes showed that piperine increases the intracellular Ca^2+^ level thus allowing the activation, through the vanilloid channel 1 (TRPV1), of Ca^2+^/calmoduline-dependent protein kinase beta (CaMKKβ) necessary for AMPK phosphorylation, which in turn enables the translocation of GLUT4 to the plasma membrane. Also, the authors observed that oral administration of piperine to Wistar rats at 0.01 and 0.1 mg/kg body weight reduced postprandial hyperglycemia [16]. Another study carried out on HT-29 cells and Caco-2 cells showed that curcumin repressed mTORC1 in HT-29 and undifferentiated Caco-2 cells, while piperine was able to enhance the mTORC1-inhibitory effect of curcumin in the same cells [13].

#### 3.3.2. In Vivo Animal and Human Studies

Studies on animal models confirmed that piperine exhibits an anti-diabetic activity, as demonstrated by a glucose-tolerance test in male Wistar rats with STZ-induced diabetes supplemented with different piperine derivatives [30], and can reduce body weight, lipid peroxidation and hepatotoxicity [18].

In a study on male Sprague Dawley rats with obesity-induced dyslipidemia, piperine supplementation at a dose of 40 mg/kg of body weight has been shown to improve lipid profile, by reducing body weight, total cholesterol, TG, and increasing HDL serum levels [89]. The mechanism of action is unknown, but it could be linked to an MC-4 agonism performed by the alkaloid. Moreover, piperine supplementation at a dose of 50 mg/kg body weight to male C57BL/6N mice with high-fat diet-induced hepatic steatosis, resulted in a significant increase in plasma adiponectin levels, together with reduced insulin, blood glucose and hepatic lipid levels [37].

Furthermore, oral administration of piperine to Wistar rats allows an increase in the Ca^2+^ levels, with consequent activation of CaMMkβ/AMPK and translocation of GLUT4 transporters, as already noted in the vitro study on preadipocyte cells [16]. Piperine has also been demonstrated to protect against β-cell dysfunction in pre-diabetic C57BL/6C mice and reduce their serum levels of LPS [39].

### 3.4. Curcumin and Piperine Combined: Mechanism of Action on Insulin Secretion/Activity and Glycemic Homeostasis

Several recent studies have demonstrated the synergic activity of curcumin and piperine in regulating glucose homeostasis when administered in combination.

#### In Vitro and In Vivo Studies

Kaur et al. (2018) [13] carried out a study on human HT-29 and Caco-2 cells where piperine has been shown to inhibit mTORC1 activity more efficiently than curcumin alone. Moreover, curcumin plus piperine resulted in greater inhibition of TNFα gene expression, thus exerting a significant anti-inflammatory activity.

The anti-inflammatory effect expressed by curcumin plus piperine is confirmed by the weight reduction in high-fat-diet mice and by reduced levels of high-fat diet-induced inflammation and reduction in IFN-γ, IL-10, IL-12 p 70, IL1β, IL6. The combination of these two compounds seems to improve metabolic syndrome in animal models, e.g., male C57BL/6 mice with high-fat diet-induced obesity [31].

In 2020, Shi et al. [28] carried out a study on a cohort of weaned piglets to assess the effects of curcumin and piperine performance, intestinal barrier function and antioxidant capacity. The co-administration of the two compounds has been shown to be more effective compared to a single high dose of curcumin, in recovering intestinal permeability and integrity and suppressing oxidative stress in this animal model.

Hoseini et al. review (2023) [34] showed that the co-administration of curcumin and piperine, independently of the dose, in patients suffering from metabolic syndrome, can restore optimal blood lipid values, with a large reduction of total cholesterol and LDL, but no effect on TG levels.

The daily co-administration of 500 mg of curcuminoids with 5 mg of piperine for 12 weeks, in 70 patients with NAFLD [38], was showed to cause a reduced hematocrit, erythrocyte sedimentation rate, serum concentration of alanine aminotransferase, total cholesterol, LDL, iron and hemoglobin, iron-binding capacity and albumin levels. Therefore, curcumin plus piperine can positively influence the lipid, glycemic and enzymatic profiles in patients with NAFLD and its advancement.

A double-blind placebo-controlled trial [40] involving 80 overweight participants with suboptimal fasting plasma glucose, which were randomized to be treated with curcumin 800 mg/day or a placebo for 8 weeks, demonstrated that the supplementation of a phytosomal preparation of curcumin containing phosphatidylserine and piperine for 56 days of treatment, reduced plasma insulin (FPI), HOMA index, TG, LDL, hepatic transaminases, γ-GT, cortisol level, and a reduction of waist circumference, blood pressure and liver steatosis index as well.

In a randomized-controlled trial conducted in T2D adult patients aged 18–65 years [12], the daily co-administration of 500 mg/day of curcuminoids with 5 mg/day of piperine for three months versus placebo caused a reduction of serum glucose, C-peptide and HbA1c, alanine aminotransferase and aspartate aminotransferase level. Therefore, the combination of curcumin and piperine may significantly improve the glycemic profile in T2D patients.

### 3.5. TLOC and Curcumin plus Piperine Supplementation

Based on the described evidence, we might postulate that the patient under examination, on a four-month treatment with a supplement containing curcumin and piperine, experienced a single and serious episode of hypoglycemia, responsible for the TLOC.

The dose taken by the patient was within the range of concentrations used in human studies to exert a pharmacological effect [85]. Moreover, the supplement in question also contained magnesium salts fatty acids (E 470) as an emulsifier, which might have promoted the formation of lipid micelles that further increased curcumin and piperine bioavailability. Considering the characteristics and pharmacokinetics of curcumin formulated with piperine, it can be assumed that after about 5 h from intake (12:30 PM was the time of meal and supplement intake and 5 PM was the time when the TLOC event was recorded), curcumin could still be sufficiently bioavailable to express its biological activity, including the hypoglycemic effects.

## 4. Conclusions

The hypoglycemic action of curcumin, mainly attributable to increased glucose uptake by cells, and the augmented secretion of insulin might have exacerbated the hypersecretion of the hormone caused by insulinoma and possibly led to hypoglycemia. We could also hypothesize that the action of curcumin on GLUT1 transporters in the brain might have contributed to a reduction of glucose available in brain tissue.

Considering the growing use of curcumin (also in combination with piperine) for the treatment of various pathologies, it would be preferable that these supplements were prescribed by competent healthcare providers.

Given the above considerations, we suggest that further research be conducted to elucidate the effects of curcumin in patients with impaired glucose metabolism and/or increased insulin secretion.

## Figures and Tables

**Table 1:** Summary of the anti-diabetic activities of curcumin and piperine as single agents and in combination.

	Curcumin	Piperine	Complex Curcumin + Piperine
In vitro studies	↓ mTORC1 signal in human intestinal epithelium cells [13]	↓ TORC1 signal in human intestinal epithelium cells [13]	↓ mTORC1 signal in human intestinal epithelium cells more efficiently than CUR alone [13]
	↑ GLUT4 translocation in skeletal muscle cells, adipocytes and hepatocytes [14,15]	↑ intracellular Ca^2+^ level with activation of CaMKKβ and consequent increase of GLUT4 translocation in L6 myotubes [16]	↓ TNFα gene expression [13]
	↑ GLUT4 expression and ↓ GLUT1 expression in hypoxic adipocytes [17]	↑ anti-diabetic activity by PPAR-gamma and ↑ insulin-sensibility in 3T3-L1 cells. [18,19]	
	↑ Akt phosphorylation [20]		
	↓pro-inflammatory cytokines in skeletal muscle cells, adipocytes and hepatocytes cells [21]		
	↓ gluconeogenesis and glycogenolysis in hepatocytes cells [22]		
	↓ G6Pase and PECK activity [22]		
	↓ mRNA expression of 11 PDE isoenzymes (PDE3B, PDE8A, PDE10A) in pancreatic islets, in dose-dependent [23]		
	↓ DPP IV in Caco2 cells [24]		
	↑ GLP-1 secretion via Ca^2+^/calmodulin-dependent Kinase pathway [25]		
	↑ insulin secretion in pancreatic islets via PDE/cAMP regulation and ↑ recovery of pancreatic islets [23]		
In vivo animal and human studies	↓ glucose serum and HbA1c levels [26,27]	↑ Ca^2+^ level with consequent translocation by APMK phosphorylation in Wistar rats [16]	↑ recovery of intestinal permeability and integrity and ↓ oxidative stress in weaned Wuzhishan piglets [28]↓
	↓ hyperlipidemia and hyperglycemia [29]	↓ body weight, hepatotoxicity and peroxidation in diabetic animal models induced by streptozotocin [18,30]	inflammation index levels and ↓ weight in high-fat-diet-RC-induced mice [31]
	↓ Inhibition of NLP3 inflammasome activation in genetic diabetes animals [32]	↑ improved lipid profile in high-fat diets induced rats [33]	↓ total cholesterol, LDL with no effect on TG in a patient with metabolic syndrome [34]
	↓ serum glucose and leptin, ↑ adiponectin and ↓insulin resistance in diet-induced diabetes models [35,36]	↑ improves insulin signal in HFD-induced hepatic steatosis, ↓plasma adiponectin and glucose levels [33,37]	↓ albumin level and improve glycemic profile in patients with NAFLD [38]
	↓ serum glucose, TG, LDL and HbA1c levels in human studies [26]	↓ β-cell-dysfunction in pre-diabetic mice and reduced LPS level [39]	↓ FPI, HOMA index, TG, LDL, hepatic transaminases, γ-GT, cortisol, blood pressure, steatosis index and waist circumference in overweight patients [40]
	↓ insulin secretion and HOMA-IR in pre-diabetic and diabetic individuals [41,42]		↓ glucose, HbA1c, C-peptide, alanine and aspartase aminotransferase in T2D patients [12]

↑: increase; ↓: reduction.

## Data Availability

Not applicable.

## Appendix A

**Table A1:** Summary of bioavailability, adverse effects, plasma peak concentrations and safety of curcumin and piperine as single agents and in combination in supplements.

	Curcumin (CUR)	Piperine (PIP)	Complex Curcumin + Piperine (CUR + PIP)
Bioavailability and pharmacokinetics	Low bioavailability Poor absorption [2] lipophilic [9] limited tissue distribution [44] single high dose causes saturation of the transport mechanism [47] rapid intestinal metabolism and clearance [44] extensive Phase I and Phase II liver transformation [45] excreted in feces as metabolites [45]	Low bioavailability Poor absorption [18] lipophilic [18] tissues distribution: liver (50%), heart, spleen, lungs, kidneys, and brain [18,63,64] subjected to oxidation, hydrogenation, methylation, glucuronidation, demethylation, sulfation and oxidation [18,65]	PIP causes higher adsorption, plasma concentration of CUR [2,4,28,44,51,60,61,62,72,73] lipophilic [9,18] ↑ bioavailability of CUR [2,4,47,48,49,65,72]
	↑ bioavailability in formulations, including micelles, nanoparticles, nano-emulsion liposomes, phospholipid complexes [48], β-casein-micelles [49], phytosomal formulations [50]	Excreted in urine and bile in form of metabolites [18,65]	↑ PIP reduces CUR glucuronidation and sulfation (red UGTs and SULTs activity) [75]
	↑ bioavailability with fresh or dried powder compared to supplements due to food matrix [53]	↑ bioavailability in the formulation including liposomes, polymeric micelles, nanoparticles, nanofibers and solid-lipid nanoparticles. [18,65]	↑ PIP enhances enzymes for the intracellular transport of nutrients (γ-glutamil transpeptidase) and inhibits drug-transporter (P-glycoprotein and CYP3A4) in enterocytes and hepatocytes [68]
	↑ intestinal microbiota enhances metabolism and biotransformation into derivates with specific biological activities [1,54] Modulation of beneficial bacteria [1,56]	↑ PIP enhances gut beneficial bacteria [66]	↑ PIP increases intestinal permeability [28]
			↑ PIP increases blood supply to the gastrointestinal tract and HCl secretion [74]
			↑ bioavailability nano-emulsion of CUR plus PIP [72]
			no significant differences were observed between the piperine-curcuminoid combination and the standard extract of single curcumin. [52]
Adverse side effect	Flatulence and gastric irritation, stimulation of bile secretion, cholangitis. No effects on glycemia [68].	Hemorrhagic stomach ulceration, mild to moderate enteritis, increased serum gonadotropins and decreased intratesticular testosterone levels. No effects on glycemia [33].	No specific study, but PIP enhanced the risk for hepatotoxicity [68,69,70,71]
Plasma peak after oral administration	1–2 h Undetectable within 12 h [46]	2–4 h Undetectable within 48 h [18,63]	No study
Safety: ADI/	0.3 mg/kg/day [67]	5 mg/day (max dose 50 mg/kg/day) [33]	No study

↑: increase; ↓: reduction.
